# *Onchocerca volvulus* as a risk factor for developing epilepsy in onchocerciasis endemic regions in the Democratic Republic of Congo: a case control study

**DOI:** 10.1186/s40249-018-0465-9

**Published:** 2018-10-08

**Authors:** Michel Mandro, Patrick Suykerbuyk, Floribert Tepage, Degratias Rossy, Francoise Ngave, Mirza Nazmul Hasan, An Hotterbeekx, Germain Mambandu, Jean Marie Kashama, Anne Laudisoit, Robert Colebunders

**Affiliations:** 1 Provincial Health Division of Ituri, Ministry of Health, Bunia, Democratic Republic of Congo; 2 Global Health Institute, University of Antwerp, Antwerp, Belgium; 3 Ministry of Health, Buta, Democratic Republic of Congo; 4 Programme national de lutte contre l’onchocercose, Kisangani, Democratic Republic of Congo; 5 Centre de Recherche en Maladies Tropicales de l’Ituri, Rethy, Democratic Republic of Congo; 6 University of Hasselt, Campus Diepenbeek, Hasselt, Belgium; 7 Office of the governor of Tshopo, Kisangani, Democratic Republic of Congo; 8 Neuropsychopathologic Centre of Mont Amba (CNPP), University of Kinshasa, Kinshasa, Democratic Republic of Congo; 9 EcoHealth Alliance, New York, USA

**Keywords:** Onchocerciasis, Epilepsy, Case control study, Risk factors, Democratic Republic of Congo

## Abstract

**Background:**

A high prevalence of epilepsy has been observed in onchocerciasis endemic areas in the Democratic Republic of Congo (DRC). With this study we aimed to investigate whether *Onchocerca volvulus* infection is a risk factor for developing epilepsy in onchocerciasis endemic regions in the DRC.

**Methods:**

Between October and December 2015, a multi-centre case control study was performed in onchocerciasis endemic health zones (HZ) in the DRC: one study site was situated in Tshopo Province in the HZ of Wanierukula (village of Salambongo) where there had been 13 annual community distributions of treatment with ivermectin (CDTI), a second was situated in Ituri Province in the HZ of Logo (village of Draju) where ivermectin had never been distributed and in the HZ of Rethy (village of Rassia) where there had been THREE CDTI annual campaigns before the study. Individuals with unprovoked convulsive epilepsy of unknown etiology were enrolled as cases (*n* = 175). Randomly selected healthy members of families without epilepsy cases from the same village and age-groups and were recruited as controls (*n* = 170).

**Results:**

Onchocerciasis associated symptoms (e.g., itching and abnormal skin) were more often present in cases compared to controls (respectively, *OR* = 2.63, 95% *CI*: 1.63–4.23, *P* <  0.0001 and *OR* = 3.23, 95% *CI*: 1.48–7.09, *P* = 0.0034). A higher number of cases was found to present with microfilariae in skin snips and with *O. volvulus* IgG4 antibodies in the blood compared to controls. Moreover, the microfilariae load in skin snips was 3–10 times higher in cases than controls.

**Conclusions:**

This case control study confirms that *O. volvulus* is a risk factor for developing epilepsy in onchocerciasis endemic regions in the DRC.

**Electronic supplementary material:**

The online version of this article (10.1186/s40249-018-0465-9) contains supplementary material, which is available to authorized users.

## Multilingual abstract

Please see Additional file 1 for translations of the abstract into five official working languages of the United Nations.

## Background

A high prevalence of epilepsy has been reported in many onchocerciasis endemic areas including in the Democratic Republic of Congo (DRC) [1–6]. In 2014, in Titule, in the Bas-Uéle Province in the DRC, 68 (2.3%) of the 2908 people who participated in an epilepsy survey were found to present with episodes of epileptic seizures. Individual risk of epilepsy was found to be associated with living close to the Bima river, a fast-flowing River where blackflies (Diptera: Simuliidae) – the vector of *Onchocerca volvulus* – oviposit and breed [5]. Between July 2014 and February 2016, several other house-to-house epilepsy prevalence surveys were carried out in the DRC in areas with a high level of onchocerciasis endemicity [6]. A high prevalence of epilepsy was observed in the villages of the three provinces investigated: 6.8–8.5% in Bas-Uele, 0.8–7.4% in Tshopo and 3.6–6.2% in Ituri [6]. These prevalences are 2–10 times higher than in non-onchocerciasis endemic regions in Africa [7]. A nested case control analysis demonstrated that before the appearance of epilepsy in individuals, compared to the same age period in controls, persons with epilepsy were approximately two times less likely (*OR* = 0.52; 95% *CI*: 0.28–0.98) to have taken ivermectin than controls [6].

In a small case control study performed in Titule, onchocerciasis suspected skin lesions were more often present in persons with epilepsy compared to controls, respectively 12/41 (29%) and 1/56 (2%) (*OR* = 20.26, 95% *CI*: 2.42–170) (*P* <  0.01) [8]. However, *O. volvulus* DNA was detected by PCR in skin snips in 26 (76%) of 34 cases and in ten (71%) of 14 controls (*P* = 0.7), and *O. volvulus* IgG4 antibodies were present in 35 (73%) of 48 cases and 15 (83%) of 18 controls (*P* = 0.5). *O. volvulus* DNA was not detected in the cerebrospinal fluid of cases [8]. The finding that in Titule there was no difference in *O. volvulus* antibodies and skin snip positivity between cases and controls is in contrast with other case control studies that found that *O. volvulus* microfilariae were more often present in cases compared to controls [9–11]. We hypothesize that this discrepancy can be explained by the fact that previous case control studies were performed prior to the introduction of mass distribution of ivermectin while in Titule annual ivermectin mass distribution has been implemented for 14 years. In the study in Titule nearly 50% of cases and controls received ivermectin 7 months before the skin snip exam [8]. We therefore performed two additional case control studies in the DRC in areas where the population had been less exposed to ivermectin.

## Methods

### Setting

**Fig. 1:**
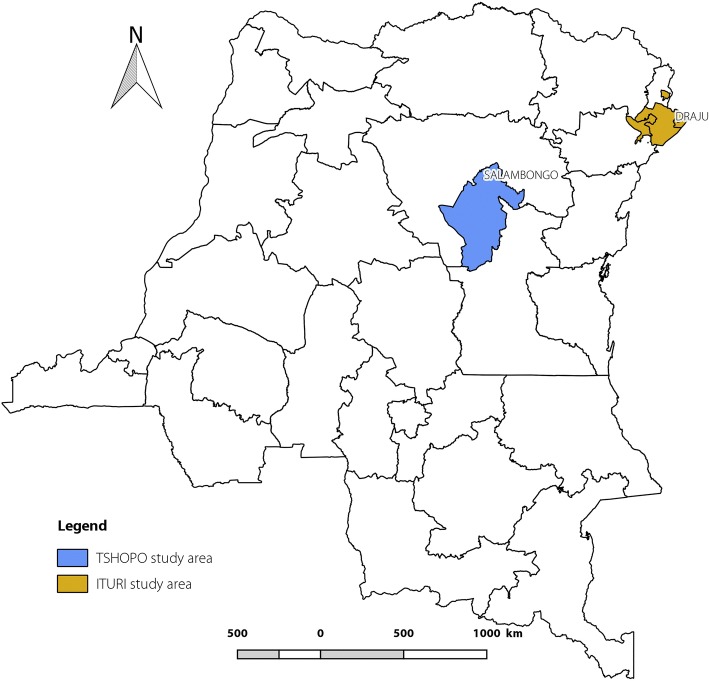
Study sites located in the Ubundu territory in Tshopo Province and the Djugu-Mahagi territories in Ituri Province, DRC

Between October and December 2015, a multicentric case control study was performed in onchocerciasis endemic health zones (HZ) in the DRC (Fig. 1): onestudy site was situated in the Tshopo Province in the HZ of Wanierukula (village of Salambongo) where there had been 13 annual community directed treatment with ivermectin (CDTI), a second in the Ituri Province in the HZ of Logo (village of Draju) where ivermectin had never been distributed and in the HZ of Rethy (village of Rassia) where there had been three CDTI campaigns before the study. House to house studies in 2015 had documented an epilepsy prevalence in Salambongo of 2.6%, in Draju 6.2% and in Rassia 3.6% [6].

### Design

One hundred seventy five individuals who developed unprovoked convulsive epilepsy of unknown etiology 12 months before the study were enrolled as cases; 170 individuals randomly selected among healthy members from the same village and age-groups were selected as controls. A standardized survey instrument was used to collect sociodemographic, clinical, and neurological data. Physical including neurological examinations were performed by a physician and a neurologist, respectively. Current infection with *O. volvulus* was assessed through detection of microfilariae in skin snip biopsies. Exposure to onchocerciasis was assessed by serology-based rapid tests (SD BIOLINE) detecting human *O. volvulus* IgG4 antibodies. Active epilepsy was defined as a patient who presented with at least 2 unprovoked seizures of unknown etiology in the last 12 months. As seizures we considered mainly tonic-clonic generalised seizures and episodes of absence, of sudden onset, and of brief duration.

### Procedures

After written informed consent was obtained, the person with epilepsy or the healthy control or their parent/guardian were interviewed in their native language by Congolese physicians (MM, KJM, GM) and local nurses using a standardized questionnaire. This questionnaire included questions about ethnicity of the mother and the father, movement of the family in the past, year of onset of the epilepsy, years of ivermectin intake, and history of febrile convulsions (defined as seizures in children < 5 years old, associated with fever, without an identified cause).

On physical examination, we assessed cases and controls for onchocerciasis nodules, skin abnormalities, vision and mental status. Height and weight were measured using a stadiometer and a digital scale and were used to calculate body mass index (BMI, kg/m^2^). Visual acuity was mainly assessed by history taking and not by a formal ophthalmological exam. A blind person was considered a person who had no light perception. The following skin lesions were considered to be onchocerciasis suspected skin lesions: a chronic scattered, pruritic, hyperpigmented papular or papulonodular eruption, dry, thickened, wrinkled skin, and spotted depigmented skin. KJM performed the neurological exams.

Blood samples were collected from all cases and controls on serobuvard filter paper (LDA22, Ploufragan, France).

A skin snip was taken from the left and right iliac crests of all subjects with a Holtz corneoscleral punch (2 mm) and stored in 90% ethanol to be tested for *O. volvulus* by an in-house PCR method (supplementary methods).

Serological tests were performed targeting *O. volvulus* IgG4 antibodies (Ov16 Standard Diagnostics, Inc., Alere SD BIOLINE, Gyeonggi-do, Republic of Korea). *Taenia solium* circulating antigen testing was performed (Cysticercosis AG Elisa, apDIa, Turnhout, Belgium) according to the manufacturers guidelines on blood eluted from the serobuvard by adding 300 μl PBS to 5 discs for each sample, with overnight incubation at 4 °C.

### Statistical analyses

Factors describing the properties of the case and control populations (weight, height, body mass index, clinical symptoms) were assessed by age-adjusted univariate models (linear regression for factors measured on a continuous scale, or else binomial logistic regression) to identify significant differences in the properties of the two groups. The relationship between parasite infection status and epilepsy status was assessed using univariate binary logistic regression models. The relationship between epilepsy status and skin biopsy/OV16 test was investigated through multiple logistic regression models where the model was adjusted by age and Ivermectin receiving status in 2014 (year before the test was conducted). Analyses were performed using Statistical Analysis System (SAS).

## Results

**Fig. 2:**
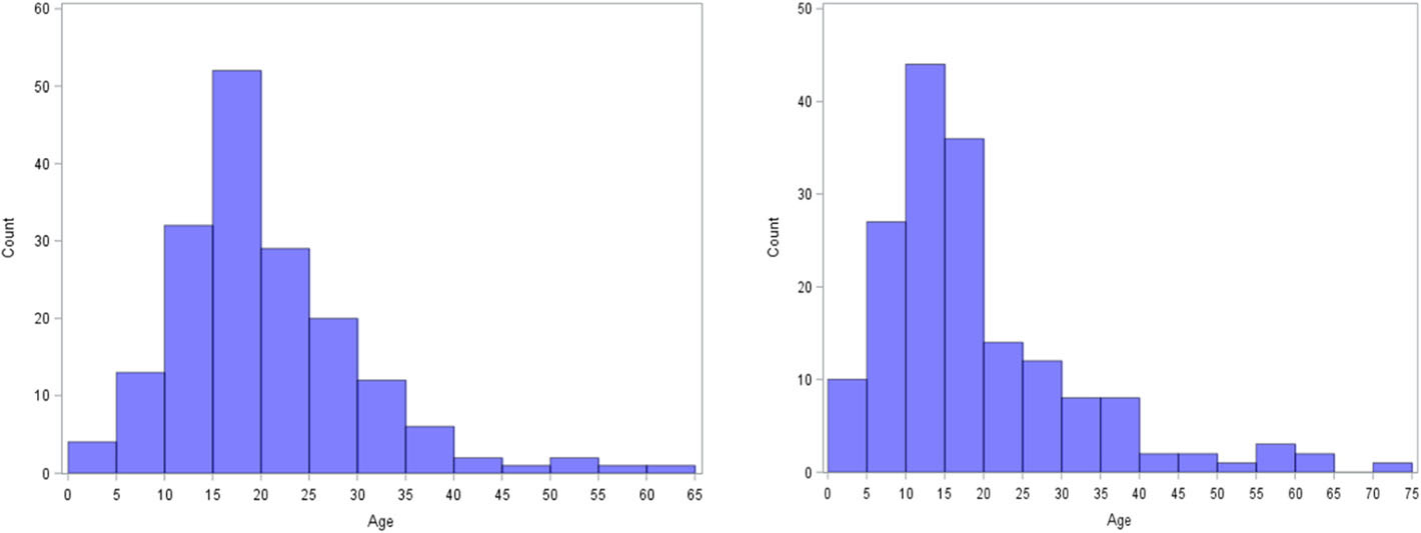
Histogram of age of the participants: Cases (left) & Controls (Right)

One hundred seventy-five cases with epilepsy (108 from Ituri and 67 from Tshopo) and 170 controls (111 from Ituri and 59 from Tshopo) were enrolled in the study (Table 1). Fifty two percent of the cases and 46% of the controls were males. Cases had an older median age (18 years) compared to the controls (15 years). The age distributions are shown in Fig. 2.

**Table 1:** Clinical characteristics of cases and controls

Clinical Characteristics	Cases (*n* = 175)	Controls (*n* = 170)	Odds Ratio (95% *CI*)^b^	*P* value
Body weight (kg) mean (*SD*)	40.93 (13.77)	38.79 (16.70)	–	0.533^a^
Height (cm) mean (*SD*)	145.50 (18.04)	140.20 (22.47)	–	0.037^a^
BMI mean (*SD*)	18.70 (3.44)	18.48 (3.83)	–	0.961^a^
Itching	78	40	2.63 (1.63–4.23)	< 0.0001
Onchocerciasis suspected skin lesions	28	10	3.23 (1.48–7.09)	0.003
Nodules	07	05	1.39 (0.41–4.66)	0.595
Burn scars	54	03	24.79 (7.55–81.34)	< 0.0001
History of febrile convulsions	14	08	2.79 (1.07–7.26)	0.035

*BMI* body mass index

^a^Age adjusted linear regression model

^b^Age adjusted binomial logistic regression model

Cases and controls were similar concerning ethnicity of the parents, place of residence and birth. Cases and controls did not differ in the distribution of main money-generating activities in their family (mainly agriculture). Cases had typically spent 1 year less in school on average (3 years, compared to 4 years in controls).

### Clinical characteristics

In 80% (140/175) of cases there was a history of tonic-clonic seizures, with drooling in 83% (146/175) and urinary incontinence in 48% (84/175); 5% (9/175) of cases had absence seizures, of which 55% (5/9) also had a history of tonic-clonic seizures. The mean age at onset of epilepsy was 11.7 years, interquartile range (third quartile-first quartile) 7 years and range (maximum-minimum) 49 years. Twenty nine percent (51/175) of cases presented with a mental disorder (an important disorder in 8.6% [15/175]) compared to 2% (3/170) of controls. Among cases with a mental disorder, 47% (24/51) had a problem with speaking; 29% (15/51) were disoriented; 51% (26/51) did not understand what was asked and forgot easily and 16% (8/51) had behavioral problems.

Onchocerciasis associated symptoms (itching and abnormal skin) and burn scars were more often present in cases compared to controls respectively (*OR* = 2.63, 95% *CI*: 1.63–4.23, *P* <  0.0001 and *OR* = 3.23, 95% *CI*: 1.48–7.09, *P* = 0.0034 and *OR* = 24.79, 95% *CI*: 7.55–81.34, *P* <  0.0001). Cases were more likely to have a history of febrile convulsions compared to controls (*OR* = 2.79, 95% *CI*: 1.07–7.26), *P* = 0.035 (Table 1).

### Laboratory results

A significantly higher number of cases compared to controls presented with microfilariae in skin snips and with *O. volvulus* IgG4 antibodies in blood. Moreover, the microfilariae load in skin snips was 3–10 times higher among cases than controls (Table 2); especially in Logo HZ where the population has never been treated with ivermectin. *T. solium* antigens were detected in two cases and none of the controls.

**Table 2:** Laboratory test results for cases and controls

	Cases			Controls			
	Draju, Logo (Ituri)	Rassia, Rethy (Ituri)	Salambongo, Wanierukula (Tshopo)	Draju, Logo (Ituri)	Rassia, Rethy (Ituri)	Salambongo, Wanierukula (Tshopo)	*P* value^e^ (a,b,c,d)
Skin biopsy test positive^f^	56% (33/59)	35% (17/49)	79% (53/67)	26% (17/65)	20% (9/45)	67% (36/54)	0.001, 0.166, 0.149, < 0.001
Mean (range) mf load^g^	31.79 (0–352.00)	9.71 (0–220.00)	27.88 (0–204.50)	2.74 (0–78.00)	3.46 (0–59.50)	18.50 (0–132.00)	< 0.001, 0.246, 0.211, < 0.001
O volvulus IgG4 antibodies	51% (30/59)	43% (21/49)	42% (28/67)	22% (14/65)	20% (9/45)	44% (26/59)	< 0.001, 0.026, 0.858, 0.002

*MF* microfilariae load

a comparison between cases and controls for Logo Health zone

b comparison between cases and controls for Rethy Health zone

c comparison between cases and controls for Wanierukula Health zone

d comparison between cases and controls for all three Health zones

^e^Fisher’s exact test was done for testing equality of two proportions and *t*-test used for testing equality of two means

^f^Skin biopsy test was considered positive if at least one microfilaria was found in either of the two skin biopsies

^g^Mean number of mf per mg skin

Skin biopsies were obtained in 339 participants of which 49% (59% [103/175] cases and 38% [62/164] controls) were positive. OV16 test was conducted for 344 participants of which 37% (45% [79/175] cases and 29% [49/169] controls) were positive. Cases were more likely to present with a positive skin test (*OR* = 2.443, 95% *CI*: 1.556–3.836, *P* = 0.0001) and to be OV16 positive (*OR* = 1.977, 95% *CI*: 1.247–3.134, *P* = 0.0037) compared to controls, holding the variables age and ivermectin intake in 2014 constant in a multiple logistic regression model. Participants who received ivermectin in 2014 were 59% (*OR* = 0.405, 95% *CI*: 0.231–0.709, *P* = 0.0015) less likely to be skin test positive compared to those who had not received ivermectin in 2014 holding the variables age and epilepsy status constant. However, ivermectin receiving status in 2014 did not have any effect on the OV16 test (*P* = 0.8012).

## Discussion

This case control study confirms that *O. volvulus* is a risk factor for developing epilepsy in onchocerciasis endemic regions in the DRC. A significant proportion of cases compared to controls were found to present with microfilariae in skin snips and to present with *O. volvulus* IgG4 antibodies in the blood compared to controls. Moreover, the microfilariae load in skin snips was 3–10 times higher in cases than controls. Differences between cases and controls were most pronounced in Draju (Logo HZ) where ivermectin was never distributed. Past ivermectin use can mask the association between epilepsy and onchocerciasis. This could explain why certain case control studies in onchocerciasis endemic regions were unable to demonstrate an association between onchocerciasis and epilepsy [12–14]. Once a person develops epilepsy his life will change dramatically compared to controls. These changes may include e.g. decreased exposure to the river and blackflies (because of the increased risk of drowning) and increased motivation to take ivermectin because of itching caused by onchocerciasis. On the other hand, healthy controls may go frequently to the river and may be less motivated to take ivermectin because they do not have itching. These factors may influence the *O. volvulus* lab results at the moment of case-control studies are performed. In Titule, in Bas Uélé Province in the DRC, with 14 years of CDTI with a therapeutic coverage around 60%, we did not find a difference in cases and controls concerning skin snip positivity [8]. This is in contrast to Draju, where ivermectin was never distributed, and 56% of cases compared to 26% of controls were skin snip positive. In Rassia, with only 3 years of CDTI, 35% of cases and 20% of controls were positive and in Salambongo, with 13 years of CDTI, 79% of cases and 67% of controls were positive. This high percentage of skin positivity among persons in Salambongo suggests a low therapeutic coverage of ivermectin. It is clear that in the latter village the CDTI programme will need to be strengthened.

Skin lesions suggesting onchocerciasis infection, itching and burn scars were also more often present in cases compared to controls.

Cases reported more often a history of febrile convulsions, confirming the observation by others that febrile convulsions may be associated with epilepsy later in life [15].

Our study has several limitations. Cases and controls were not individually matched by age and cases were slightly older than controls. Although *O. volvulus*-infection rates increase by age, a 3 year median age difference cannot explain the large difference in *O. volvulus*-infection rate between cases and controls. *T. solium* antigen serological testing was the only test done to identify other causes of epilepsy. Moreover, normally serum samples are tested undiluted, while for this study only filter paper samples were available, introducing a dilution of the tested samples, leading to a decreased test sensitivity. Therefore, the results of the *T. solium* antigen tests need to be interpreted with great caution.

Our study confirms the findings of other epidemiological studies that *O. volvulus* directly or indirectly act as a trigger for developing epilepsy in onchocerciasis endemic regions [16]. However, the mechanism how an *O. volvulus* infection could lead to epilepsy remains unclear. Indeed, *O. volvulus* microfilariae are only exceptionally found in the central nervous system [17]. In Tanzania and in the DRC, PCR tests on cerebrospinal fluid (CSF) of patients with nodding syndrome and epilepsy with other types of seizures failed to identify *O. volvulus* DNA [8, 17]. A recent study suggested that the neurotoxic anti-leiomodin-1 antibodies, which cross-react with *O. volvulus*-specific proteins could play a role in causing nodding syndrome [18]. In another study in Uganda, more serum antibodies against voltage gated potassium channel-complex proteins were detected in patients with nodding syndrome compared to controls [19]. However, whether these autoantibodies are the cause of the nodding syndrome or the consequence of damage to the neurons caused by another mechanism remains to be investigated [16].

## Conclusions

This case control study confirms the growing body of literature that *O. volvulus* is a risk factor for developing epilepsy in onchocerciasis endemic regions in Africa.

## Additional file

Additional file 1: Multilingual abstracts in the five official working languages of the United Nations. (PDF 336 kb)

## Abbreviations

BMI: Body mass index
CDTI: Community distributions of treatment with ivermectin
*CI*: Confidence interval
CSF: Cerebrospinal fluid
DRC: Democratic Republic of Congo
HZ: Health zones
mf: Microfilaria
*OR*: Odds ratio
