# Supplementary material for: Onchocerca volvulus as a risk factor for developing epilepsy in onchocerciasis endemic regions in the Democratic Republic of Congo: a case control study
Source: Infect Dis Poverty. 2018 Oct 8;7:79. doi: 10.1186/s40249-018-0465-9 (PMC6174565; doi:10.1186/s40249-018-0465-9)
Supplement: Supplementary file 1 — Multilingual abstracts in the five official working languages of the United Nations. (PDF 336 kb) [file 40249_2018_465_MOESM1_ESM.pdf]

Translation of the abstract into the five official working languages of the United Nations

كلابية الذنب المتلوية كعامل خطر لتقدم الصرع في المناطق الموبوءة به في جمهورية الكونغو الديمقراطية: دراسة مراقبة حالة

ميشال ماندرو، باتريك سويكيربويك، فلوريبرت تيباجي، ديجراتياس روسي، فرنسواز نجيف، ميرزا نزمول حسن، آن هوتيربيككس، جرمان مامباندو، جان ماري كاشاما، آن لوديسويت، وروبرت كوليبونديرس

الملخص
الخلفية: لوحظ تفشي الإصابة بمرض الصرع في المناطق الموبوءة بداء كلابية الذنب الملتوية في جمهورية الكونجو الديمقراطية. وقد كان هدفنا عبر هذه الدراسة- هو التحقيق في احتمالية كون داء كلابية الذنبعامل خطر لتقدم مرض الصرع في المناطق الموبوءة به في جمهورية الكونغو الديمقراطية.
الطُرُق: أجريت -في الفترة بين أكتوبر/تشرين الأول وديسمبر/كانون الأول من عام 2015- دراسة مراقبة حالة متعددة المراكز في المناطق الصحية المستوطن فيها داء كلابية الذنب (HZ) من جمهورية الكونغو الديمقراطية: وقد كانت مقاطعة تشوبو -الواقعة في منطقة وانيريوكولا الصحية- (قرية سلامبونجو) أحد مواقع الدراسة؛ حيث كان هناك 13 توزيعًا محليًا سنويًا لدواء إيفيرمكتين. كما كانت مقاطعة إيتوري الواقعة في منطقة لوجو الصحية (قرية دراجو) موقعًا آخر للدراسة؛ حيث لم يُوزع الإيفرمكتين هناك أبدًا، بالإضافة إلى مقاطعة ريتي الصحية (قرية راسيا)؛ حيث نُفذت ثلاث حملات للتوزيع المحلي السنوي لدواء الإيفيرمكتين قبل إجراء الدراسة.سُجل الأشخاص المصابون بالصرع الاختلاجي غير معروف الأسباب كحالات (n = 175). واختيرعشوائيًا عدد من الأفراد الأصحاء -من عائلات ليس لديها أي تاريخ بالإصابة بالمرض- وينتمون لنفس القرية والفئة العمرية؛ ليتم تجنيدهم كأفراد مراقبة (n = 170).
النتائج: ظهرت أعراض داء كلابية الذنب (مثل: الحكة والبشرة غير الطبيعية) في الحالات مقارنة بعناصر المراقبة بنسب (على التوالي، OR = 2.63, 95% CI: 1.63–4.23, P < 0.0001 and OR = 3.23, 95% CI: 1.48–7.09, P = 0.0034). كما أظهر عدد أكبر من الحالات أعراض مثل ميكروفيلاريا في القصاصات الجلدية بالإضافة إلى داء كلابية الذنب وأجسام مضادة في الدم مقارنة بعناصر المراقبة. وعلاوة على ذلك، كانت نسبة الميكروفيلاريا في القصاصات الجلدية تمثل 3-10 مرات أعلى في الحالات مقارنة بعناصر المراقبة.
الاستنتاجات: دراسة مراقبة الحالة هذه تؤكد أن داء كلابية الذنب يُمثل عامل خطر لتقدم الصرع في المناطق الموبوءة به في جمهورية الكونغو الديمقراطية.

Translated from English version into Arabic by Maher Abassi and Amal Imam, through

TRANSLATORS
WITHOUT BORDERS

**盘尾丝虫是造成癫痫的风险因素 :在刚果民主共和国盘尾丝虫病流行区开展的一项病例对照研究**

Michel Mandro, Patrick Suykerbuyk, Floribert Tepage, Degratias Rossy, Francoise Ngave, Mirza Nazmul Hasan, An Hotterbeekx, Germain Mambandu, Jean Marie Kashama, Anne Laudisoit and Robert Colebunders

**摘要：**
**引言：**在刚果民主共和国(DRC)盘尾丝虫病流行区，癫痫的流行率也较高。本研究旨在探讨在该地区盘尾丝虫感染是否为造成癫痫的风险因素。
**方法：**2015 年 10 月至 12 月，在 DRC 盘尾丝虫病地方病卫生区（HZ）开展了一项多中心病例对照研究。一个研究点位于 Tshopo 省的 Wanierukula HZ（Salambongo 村），该地区已接受 13 次年度社区分配伊维菌素治疗（CDTI）。第二个研究点位于伊图里省，其中 Logo HZ (Draju 村)未分发过伊维菌素，而 Rethy HZ(Rassia 村)在开展此次研究前曾三次大规模

地实施年度 CDTI。该研究把罹患未知病因的无意识惊厥性癫痫患者纳入病例组（$n = 175$），随机选择同村中的从未有过癫痫病例的健康家庭里同年龄段的成员的作为对照组（$n = 170$）。
**结果：**与对照组相比，病例组中盘尾丝虫病的相关症状（例如瘙痒和皮肤异常）更为常见（分别为 $OR = 3.23$，95％ $CI$：1.48–7.09，$P = 0.0034$ 和 $OR = 2.63$，95％$CI$：1.63-4.23，$P < 0.0001$）。在病例组中，皮片中出现微丝蚴，血液中的盘尾丝虫 IgG4 抗体的病例数更高。而且，病例组中的皮片微丝蚴载量比对照组高 3–10 倍。
**结论：**该病例对照研究证实，在 DRC 盘尾丝虫病流行区，盘尾丝虫是造成癫痫的风险因素。

Translated from English version into Chinese by Qin-Yun Chen, edited by Jin Chen

***Onchocerca volvulus*** **en tant que facteur de risque de développement de l'épilepsie dans les zones d'endémie de la République démocratique du Congo : étude de cas**

Michel Mandro, Patrick Suykerbuyk, Floribert Tepage, Degratias Rossy, Francoise Ngave, Mirza Nazmul Hasan, An Hotterbeekx, Germain Mambandu, Jean Marie Kashama, Anne Laudisoit et Robert Colebunders

**Résumé**
**Contexte :** Une forte prévalence de l'épilepsie a été observée dans les zones d'onchocercose endémique en République démocratique du Congo (RDC). Avec cette étude, nous avons cherché à déterminer si l'infestation par *Onchocerca volvulus* était un facteur de risque de développement de l'épilepsie dans les régions d'onchocercose endémique de RDC.
**Méthodes :** Entre octobre et décembre 2015, une étude cas-témoins multicentrique a été réalisée dans les zones sanitaires (ZS) d'onchocercose endémique en RDC : un site d'étude était situé dans la province de Tshopo, dans la ZS de Wanie-Rukula (village de Salambongo) où avaient eu lieu 13 distributions de traitements à l'ivermectine sous directives communautaires (TIDC), un deuxième site dans la province d'Ituri, dans la ZS de Logo (village de Draju) où l'ivermectine n'avait jamais été distribuée, et un troisième dans la ZS de Rethy (village de Rassia) où trois campagnes annuelles de TIDC s'étaient déroulées avant l'étude. Nous avons inclus dans notre étude les sujets souffrant d'une épilepsie convulsive non provoquée d'étiologie inconnue ($n = 175$). Des personnes en bonne santé d'âge correspondant, choisies au hasard dans des familles sans cas d'épilepsie appartenant au même village, ont été recrutées comme témoins ($n = 170$).
**Résultats :** Les symptômes associés à l'onchocercose (p. ex. démangeaisons et lésions cutanées) étaient plus fréquents parmi les cas que parmi les témoins (respectivement, $OR = 2{,}63$, $IC$ à 95 % de 1,63 à 4,23, $P < 0{,}0001$ et $OR = 3{,}23$, $IC$ à 95 % de 1,48 à 7,09, $P = 0{,}0034$). Il s'est avéré qu'un plus grand nombre de cas que de témoins présentaient des microfilaires dans les replis de la peau ainsi que des anticorps IgG4 visant *O. volvulus* dans le sang. De plus, la présence de microfilaires dans les biopsies cutanées exsangues était de 3 à 10 fois plus importante parmi les cas que parmi les témoins.
**Conclusions :** Cette étude cas-témoin confirme qu'*O. volvulus* est un facteur de risque de développement de l'épilepsie dans les régions d'onchocercose endémique de RDC.

Translated from English version into French by Louis Gauvreau and Suzanne Assenat, through

***Onchocerca volvulus* как фактор риска развития эпилепсии в эндемичных по онкоцеркозу регионах Демократической Республики Конго: исследование по методу «случай-контроль»**

Мишель Мандро, Патрик Сьюкербьюк, Флорибер Тепаж, Дегратиас Росси, Франсуаза Нгаве, Мирза Назмуль Хасан, Эн Хоттербикс, Жермен Мамбанду, Жан Мари Кашама, Анн Лаудисуа и Роберт Коулбандерс

**Аннотация**

**Справочная информация:** В Демократической Республике Конго (ДРК), в эндемичных очагах по онхоцеркозу, отмечена высокая частота случаев эпилепсии. Проводя это исследование, мы стремились выяснить, является ли инфекция, вызываемая *Onchocerca volvulus*, фактором риска развития эпилепсии в эндемичных регионах по онхоцеркозу в ДРК.

**Методы:** На протяжении периода с октября по декабрь 2015 года было проведено многоцентровое исследование по методу «случай-контроль» в зонах здоровья (ЗЗ), эндемичных по онхоцеркозу в ДРК: один центр исследования находился в провинции Чопо в ЗЗ Ваньерюкюла (в деревне Саламбонго), где были проведены 13 ежегодных общинных курсов лечения ивермектином (ОКЛИ), второй был расположен в провинции Итури в ЗЗ Лого (в деревне Дражю), где ивермектин никогда не применялся, а также в ЗЗ Рети (в деревне Рассиа), где перед началом исследования были проведены ТРИ ежегодных кампании ОКЛИ. В качестве субъектов исследования («случаев»)были отобраны люди с неспровоцированной судорожной эпилепсией неизвестной этиологии ($n = 175$). Отобранные случайным образом здоровые члены семей без заболевания эпилепсией из той же деревни, принадлежащих к таким же возрастным группам, были включены в качестве контрольных лиц ($n = 170$).

**Результаты:** Симптомы, связанные с онкоцеркозом (например, зуд и кожная аномалия) более часто встречались у субъектов исследования, чем у членов контрольной группы (соответственно, *OR* = 2,63; 95% *CI*: 1,63–4,23, $P < 0,0001$ и *OR* = 3,23; 95% *CI*: 1,48–7,09; $P = 0,0034$). У большего количества субъектов исследования были обнаружены микрофилярии в образцах кожи и *антитела IgG4 к O. volvulus в крови* по сравнению с членами контрольной группы. Более того, уровень микрофилярий в биоптатах кожи был в 3—10 раз выше у субъектов исследования, чем у лиц из контрольной группы.

**Выводы:** Результаты данного исследования по методу «случай-контроль» подтверждают, что *O. volvulus* является фактором риска развития эпилепсии в эндемичных по онхоцеркозу регионах ДРК.

Translated from English version into Russian by Ann Nosova and Liudmila Tomanek, through

**El *Onchocerca volvulus* como factor de riesgo para la aparición de la epilepsia en regiones endémicas de oncocercosis en la República Democrática del Congo: un estudio de control de casos**

Michel Mandro, Patrick Suykerbuyk, Floribert Tepage, Degratias Rossy, Francoise Ngave, Mirza Nazmul Hasan, An Hotterbeekx, Germain Mambandu, Jean Marie Kashama, Anne Laudisoit y Robert Colebunders

**Resumen**

**Antecedentes:** Se ha observado una alta prevalencia de epilepsia en zonas endémicas de oncocercosis en la República Democrática del Congo (RD Congo). Este estudio tiene por objeto investigar si la infección por *Onchocerca volvulus* es un factor de riesgo para la aparición de la epilepsia en regiones endémicas de oncocercosis en la RD Congo.

**Métodos:** Entre octubre y diciembre de 2015 se realizó un estudio multicéntrico de control de casos en zonas de salud (Health Zone, HZ) de oncocercosis endémica en la RD Congo: un sitio del estudio estaba situado en la provincia de Tshopo en la HZ de Waneirukula (población de Salamongo) donde se habían hecho trece distribuciones anuales comunitarias de tratamiento con ivermectina (CDTI); otro sitio estaba situado en la provincia de Ituri, en la HZ de Logo (población de Draju) donde nunca se había distribuido ivermectina y en la HZ de Rethy (población de Rassia) donde se habían hecho TRES campañas anuales de CDTI antes del estudio. Se incorporaron al estudio como casos pacientes con epilepsia convulsiva no provocada de etiología desconocida ($n$ = 175). También se incorporaron al estudio como controles miembros de familias saludables, sin casos de epilepsia, que fueron seleccionados en forma aleatoria en el mismo pueblo y en los mismos grupos etarios.($n$ = 170).

**Resultados:** Los síntomas relacionados con la oncocercosis (como prurito y piel anormal) se presentaron más frecuentemente en los casos, frente a los controles (respectivamente, *OR* = 2.63, 95% *CI*: 1.63–4.23, $P < 0.0001$ y *OR* = 3.23, 95% *CI*: 1.48–7.09, $P = 0.0034$). Se encontraron más casos que se presentaban con microfiliaria en pellizcos cutáneos y con anticuerpos IgG4 de *O. volvulus* en la sangre comparados con los controles. Además, la carga de microfiliaria en los pellizcos cutáneos fue entre tres y diez veces mayor en los casos que en los controles.

**Conclusiones:** Este estudio de control de casos confirma que el *O. volvulus* es un factor de riesgo para el desarrollo de la epilepsia en regiones endémicas de oncocercosis en la RD Congo.

Translated from English version into Spanish by Diana Kreimer and Carlos González-Rivera, CT, through

TRANSLATORS WITHOUT BORDERS
